# The Role of CD4^+^T Cells in Nonalcoholic Steatohepatitis and Hepatocellular Carcinoma

**DOI:** 10.3390/ijms25136895

**Published:** 2024-06-23

**Authors:** Yadi Miao, Ziyong Li, Juan Feng, Xia Lei, Juanjuan Shan, Cheng Qian, Jiatao Li

**Affiliations:** 1 School of Medicine, Chongqing University, Chongqing 400030, China; 2 Center for Precision Medicine of Cancer, Chongqing Key Laboratory of Translational Research for Cancer Metastasis and Individualized Treatment, Chongqing University Cancer Hospital, Chongqing 400030, China

**Keywords:** nonalcoholic steatohepatitis, hepatocellular carcinoma, CD4^+^T cell, immunotherapy

## Abstract

Hepatocellular carcinoma (HCC) has become the fourth leading cause of cancer-related deaths worldwide; annually, approximately 830,000 deaths related to liver cancer are diagnosed globally. Since early-stage HCC is clinically asymptomatic, traditional treatment modalities, including surgical ablation, are usually not applicable or result in recurrence. Immunotherapy, particularly immune checkpoint blockade (ICB), provides new hope for cancer therapy; however, immune evasion mechanisms counteract its efficiency. In addition to viral exposure and alcohol addiction, nonalcoholic steatohepatitis (NASH) has become a major cause of HCC. Owing to NASH-related aberrant T cell activation causing tissue damage that leads to impaired immune surveillance, NASH-associated HCC patients respond much less efficiently to ICB treatment than do patients with other etiologies. In addition, abnormal inflammation contributes to NASH progression and NASH–HCC transition, as well as to HCC immune evasion. Therefore, uncovering the detailed mechanism governing how NASH-associated immune cells contribute to NASH progression would benefit HCC prevention and improve HCC immunotherapy efficiency. In the following review, we focused our attention on summarizing the current knowledge of the role of CD4^+^T cells in NASH and HCC progression, and discuss potential therapeutic strategies involving the targeting of CD4^+^T cells for the treatment of NASH and HCC.

## 1. Introduction

According to the GLOBOCAN 2020 report by the World Health Organization (WHO) and the International Agency for Research on Cancer, liver cancer is a major global public health problem and has become the fourth leading cause of cancer-related deaths. Approximately 906,000 new liver cancer cases and 830,000 deaths related to cancer liver are diagnosed globally each year, a significant indication of the seriousness of this disease and its impact on global health [1,2,3]. As the most prevalent primary liver cancer, hepatocellular carcinoma (HCC) accounts for 75% of liver cancer mortality [2]. The leading cause of HCC includes viral factors, such as hepatitis B virus (HBV) and chronic hepatitis C virus infections, as well as non-viral factors, such as alcohol addiction, exposure to dietary toxins, such as aflatoxins, and nonalcoholic fatty liver disease (NAFLD). NAFLD is constituted by excess fat in hepatocytes, which is strongly associated with obesity, type 2 diabetes, and hypertension [4,5,6]. Nonalcoholic steatohepatitis (NASH) is a more severe disorder that is derived from NAFLD and is also a key risk factor for the development of NASH-associated cirrhosis and HCC. Genetic factors, lipid overload, toxicity, oxidative stress, and inflammation have all been implicated in the development and progression of NASH. Studies have supported a strong mechanistic link between the NASH microenvironment and HCC development [7]. The prevalence of NASH in the United States accounts for about 3–4% of the entire population, and the incidence of NAFLD and NASH is increasing each year [8]. In addition, the development of viral treatments means that viral infections, including hepatitis C virus and hepatitis B virus, can be partially treated, while effective treatments are currently unavailable for NASH and NASH-associated HCC. Thus, NASH has surpassed viral infection as the leading cause of HCC in Western countries, highlighting the urgency to uncover the mechanism of NASH occurrence and NASH–HCC transition, with the goal of developing new therapeutic strategies [9,10].

**Figure 1:**
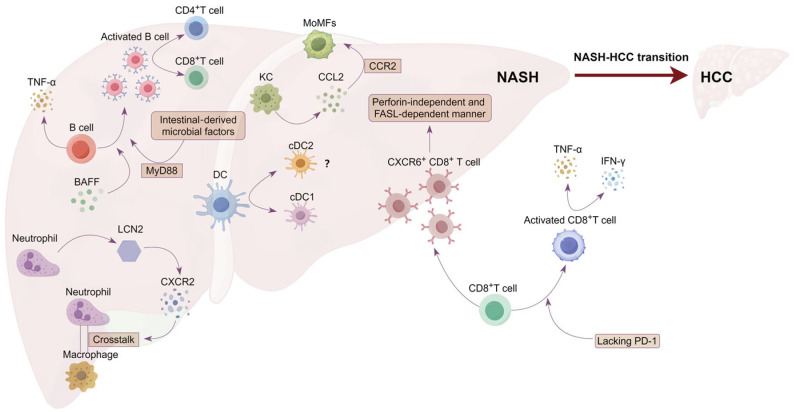
The role of B cells, DCs, neutrophils, KCs, and CD8^+^T cells in NASH progression. B-cell-activating factor (BAFF) is involved in B cell activation, and intestinal-derived microbial factors activate B cells in the liver in a manner dependent on myeloid differentiation primary response protein 88 (MyD88). Activated B cells promote NASH progression by regulating CD4^+^T cell and CD8^+^T cell activation and the secretion of TNF-α. Both conventional DC1s (cDC1s) and conventional DC2s (cDC2s) are present in the liver and accumulate during NASH progression. cDC1s have been shown to promote liver damage in mice. Neutrophil-derived lipid carrier protein 2 (LCN2) exacerbates steatohepatitis by inducing CXC motif chemokine receptor 2 (CXCR2) expression to promote crosstalk between neutrophils and liver macrophages. Activated Kuffer cells (KCs) promote the recruitment of monocyte-derived macrophages (MoMFs) in a C manner dependent on –C chemokine receptor type 2 (CCR2) by secreting C-C motif ligand 2 (CCL2). MoMFs further amplify the inflammatory response. CD8^+^T cells, especially CXCR6^+^ CD8^+^T cells, directly induce hepatocyte injury and NASH–HCC transition in a perforin-independent and FASL-independent manner. Mice lacking PD-1 showed an increased incidence of liver cancer, which may be due to increased CD8^+^T cell activation as well as cytokine expression (IFN-γ, TNF-α).

At present, treatment options for HCC include surgical resection, local ablative therapy, liver transplantation, targeted therapy, immunotherapy, and chemotherapy; however, no approved therapy exists for the treatment of NASH. In general, these treatment modalities are mainly used in combination in clinical practice, and the specific choice depends on the stage of the tumor, as well as on the patient’s physical condition and liver function status [11]. Since patients with early-stage HCC have no obvious clinical symptoms, most newly diagnosed patients are found to be in the middle and late stages of cancer and thus cannot be transplanted, resected, or even treated locally. Chemoradiotherapy and targeted therapy are often used for late-stage HCC, but drug resistance usually occurs in such cases [12,13]. T-cell-based immunotherapy, in particular the use of immune checkpoint inhibitors (ICIs), has shown great potential in mediating tumor regression in HCC treatment. Based on good clinical results, ICIs have been approved by the FDA as a new first-line treatment for HCC [14], highlighting the importance of immune surveillance in HCC progression. However, it was reported that compared to other etiologies, NASH-induced HCC patients responded much less efficiently to treatment with ICIs, probably owing to NASH-related aberrant T cell activation causing tissue damage that leads to impaired immune surveillance [15]. Therefore, uncovering the underlying mechanism governing how immune cells interact with hepatocytes in NASH progression is crucial to the development of new immunotherapy for HCC. Both the innate immune response and adaptive immunity contribute to NASH-associated inflammation [16]. For example, innate immune cells, including dendritic cells (DCs), neutrophils, Kuffer cells (KCs), and B cells, could promote liver inflammation to promote NASH progression (Figure 1) [15,17,18,19,20,21,22,23]. Preclinical and human studies have implicated various subsets of conventional and innate-like T cells in the onset and progression of NASH and HCC [24]. CD8^+^T cells, particularly CXCR6^+^CD8^+^T cells, directly induce hepatocyte injury and NASH–HCC transformation in a perforin-independent and Fas ligand (FASL)-dependent manner. The function of the innate immune and adaptive immune cells in NASH progression is shown in Figure 1.

The role of CD4^+^T cells in supporting the effector function of CD8^+^T cells and innate immunity was described in mouse cancer models as early as 1982 [25]. Meanwhile, it has been shown that CD4^+^T cells play a non-redundant role in maintaining and sustaining antitumor CD8^+^CTL responses [26,27]. Of late, in addition to playing an adjuvant role of as helper immune cells in promoting cytotoxic CD8^+^T lymphocytes as well as the innate immune response, the cytotoxic role of CD4^+^T cells in cancer has drawn increasing attention [28]. Nevertheless, the critical role of CD4^+^T cells in orchestrating the antitumor response is still eclipsed by the clinical success of CD8^+^T cell-based immunotherapies. The fundamental role of CD4^+^T cells in carrying out direct cytotoxicity against cancer cells independent of CD8^+^T cells was first reported by Linnemann et al., who found that human melanomas contained neoepitopes recognized only by CD4^+^T cells. Their findings were followed by a report that, in a study, immunogenic tumor neo-antigens induced a CD4^+^T cell response but not the expected CD8^+^T cell response [29,30]. These findings were further confirmed by Edward Seung et al., who showed that a trispecific antibody targeting HER2 and T cells inhibits breast cancer growth via CD4^+^T cells but not via CD8^+^T cells [31]. In another report, David Y Oh et al. used single-cell RNA sequencing to show that multiple cytotoxic CD4^+^T cell states in bladder cancer can kill autologous tumors in an MHC class II-dependent manner [32]. It is now widely accepted that CD4^+^T cells are antitumor effector cells in their own right, corresponding to their long-known role as central players in and coordinators of innate and antigen-specific immune response [33]. The role of CD4^+^T cells in NASH initiation and progression, NASH–HCC transition, and HCC progression has been reported during HCC development. However, further mechanistic investigation is still required before clinical application via CD4^+^T cell manipulation in NASH or HCC treatment. Therefore, in this review, we focused our attention on summarizing the current knowledge of the role of CD4^+^T cells in NASH and HCC progression, and discuss potential therapeutic strategies involving the targeting of CD4^+^T cells for the treatment of NASH and HCC.

## 2. The Role of CD4^+^T Cells in NASH Progression

CD4^+^T cells can be classified into Th effector cells and regulatory T cells (Tregs). The balance between them plays an important role in maintaining tissue-immune homeostasis. Th cells promote the immune response to neoantigens derived from pathogens, allergens, and cancer cells; in comparison, Tregs suppress the function of Th cells through the secretion of immune suppressive cytokines, including IL-10 and IL-33. Based on the expression of transcriptional factors and cytokines, effector Th cells can be further divided into Th1, Th2, Th17, and Th22 subsets [34]. Lymphocyte infiltration, one of the histological features of NASH, parallels worsening parenchymal injury and lobular inflammation [21]. Recruitment of CD4^+^T cells in NASH livers has been observed in both experimental mouse models and human NASH patients. In a study, the depletion of total CD4^+^T cells reduced the inflammation observed in NASH and ameliorated NAFLD activity scores and liver fibrosis in humanized NASH mice, indicating the functional role of CD4^+^T cells in NASH development [21,35,36]. Increasing evidence has shown that different subsets of CD4^+^T cells may play various roles in NASH progression.

***Th1*:** Th1 cells comprise a subgroup of CD4^+^T cells that play an important role in the adaptive immune system, especially in defense against intracellular pathogens [37]. Th1 cells are characterized by the expression of transcriptional factor T-bet and the production of pro-inflammatory cytokines such as interferon-γ (IFN-γ),tumor necrosis factor α (TNF-α) and IL-2 [38]. In addition to their role in eliminating pathogens and tumor-associated neoantigens, Th1 cells are also implicated in the progression of tissue damage, including adipose tissue inflammation associated with obesity [39], and impair neoangiogenesis in the ischemic tissue of individuals with type 2 diabetes through the secretion of IFN-γ and TNF-α [40]. 

Using samples from NASH and NAFLD patients, Monika Rau et al. demonstrated in their study that higher frequencies of IFN-γ^+^Th1 cells were detected among total CD4^+^T cells in the peripheral blood of NASH patients compared to healthy controls. Th1 cells are also enriched in the NASH liver compared to the blood [19]. In another study, Maria Eugenia Inzaugarat found that NASH patients showed increased numbers of CD4^+^ cells, and the number of IFN-γ-producing CD4^+^ and CD8^+^T cells was also found to be increased in the peripheral blood of NASH patients [41]. The authors of another study used a liver tissue gene expression profile and found that inflammatory and immune response genes were upregulated in NASH patients, with the most notable changes being found in genes encoding chemokines/chemokine receptors involved in leukocyte recruitment, as well as cytokines involved in Th1 polarization, such as IFN-γ, TNF-α, and IL-1β [42].

In a concanavalin A hepatitis plus choline-deficient diet mouse model, the expression of Th1-related cytokines, including IFN-γ, TNF-α, and IL-12, as well as Th1 marker transcriptional factors T-bet and STAT4, was dramatically elevated compared with control-fed mice [43]. The MCD diet (methionine choline-deficient)-induced NASH mouse model was found to be able to reproduce all phases of human NASH progression. By using an MCD diet-induced NASH mouse model, Simona Rolla et al. showed that CD4^+^T cell infiltration in the liver was significantly elevated as early as week 1 post-MCD diet; in comparison, IFN-γ^+^Th1 increased most significantly during week 8 [44]. Furthermore, employing IFN-γ-deficient mice, Xiao-Yu Luo et al. utilized an MCD-HF diet-induced NASH model to show that liver fibrosis was significantly reduced in IFN-γ knockout mice. Mechanically, IFN-γ deficiency inhibits Kuffer cells’ inflammatory response to express fibrosis-related cytokines, including TNF-α [45]. As IFN-γ can also induce cell cycle arrest and hepatocyte apoptosis, Th1 may also accelerate the evolution of NASH by directly acting on hepatocytes [46,47,48,49].

The effect of tumor necrosis factor-α (TNF-α), another important pro-inflammatory cytokine secreted by Th1 cells, on the pathogenesis of hepatic steatohepatitis has also been widely established to promote NASH progression [50]. In patients with steatohepatitis, TNF-α levels are elevated in serum, hepatic tissue, and adipose tissue [51,52,53,54,55], and this level increases along with steatosis progression to NASH [54,56]. TNF-α has been implicated as a major pathogenic driver of NAFLD. In a high-fat diet (HFD)-induced mouse model of NAFLD, inhibition of TNF-receptor-1 significantly reduced liver steatosis, improved insulin resistance, decreased liver injury, and decreased liver fibrosis, suggesting TNFR1 as a promising approach for NAFLD treatment [57]. Notably, TNF-α is not just secreted by Th1 cells; it is also abundantly expressed by macrophages, CD8^+^T cells, and other myeloid cells [58].

Collectively, both NASH human patient samples and NASH mouse models support the hypothesis that Th1 accelerates the initiation and progression of NASH, which may be due to the secretion of cytokines, such as IFN-γ and TNF-α.

***Th2*:** Th2 cells are characterized by the expression of transcriptional factor GATA3 and the secretion of cytokines, including IL4, IL5, IL10, and IL13 [59]. Th2 cells exert both anti-inflammatory and pro-inflammatory effects. In host defense, the secretion of IL4, IL5, and IL13 by Th2 cells triggers an inflammatory response to provide defense against pathogens and allergens. The findings of other studies also indicate the non-redundant role of Th2 cells in tissue repair via the promotion of the resolution of inflammation and the restoration of tissue homeostasis [60,61].

Examining NASH and NAFLD patients, Monika Rau reported that there are more IL4^+^Th2 cells in the peripheral blood of both of these patients compared to healthy control donors. The proportion of Th2 cells out of the total number of CD4^+^T cells is also more highly enriched in the liver tissue than in the blood of NASH and NAFLD patients, with no difference observed between these patients, suggesting an important role of Th2 in NASH and NAFLD progression [36].

NASH patients show increased levels of IL-13, and its receptor IL-13 Rα2 is also highly expressed in hepatic stellate cells. In one study, the targeting of IL13Ra2 HSCs in NASH rats attenuated liver fibrosis without inducing organ toxicity. Since Th2 expresses high levels of IL13, and its proportion was found to increase in the liver and blood of NASH patients, this finding suggests that Th2-derived IL13 may promote NASH progression by interacting with IL-13Rα2-positive hepatic stellate cells [62]. IL-33 expression is found in hepatic stellate cells in both NASH patients and mouse liver tissue, and in a study, IL33 expression was found to be elevated in NASH mouse livers. Treatment with IL33 attenuated diet-induced hepatic steatosis, and this effect may be due to the promotional effect of IL33 on Th2 response and M2 macrophage activation in mice exposed to a high-fat diet or an MCD diet [63,64]. Collectively, even though the available evidence indicates the importance of Th2-related cytokines in NASH progression in both patient samples and mouse models, the direct effect of Th2 on NASH progression requires further investigation.

***Th17*:** Th17 cells comprise an important subgroup of CD4^+^T cells; their differentiation is controlled by transcription factors and epigenetic modification [65]. Th17 cells are characterized by the expression of transcriptional factors RORγt and STAT3, as well as by the secretion of cytokine IL-17 [66,67]. Th17 cells are often thought to be pro-inflammatory and play a key role in the pathogenesis of inflammatory responses and autoimmune diseases [68,69]. The livers of patients with NASH show an increased number of Th17 cells compared to those of patients with NAFLD, suggesting a role of Th17 in NAFLD–NASH progression [36].

The IL-17 family consists of six structurally related cytokines: IL-17A, IL17B, IL-17C, IL-17D, IL-17E, and IL-17F. Most IL-17 subtypes are essential for acute and chronic inflammation mediated by innate and adaptive immunity [70]. Using gene knockout mice, Daniel A Giles et al. demonstrated that the knockout of *IL17A*, *IL17RA*, and *IL17F* exhibited significant protective effects against hepatocellular damage compared to WT controls in methionine- and choline-deficient diet (MCDD)-induced NASH mouse models. Mechanistically, the MCDD diet augmented hepatic IL17RA expression and increased the hepatic infiltration of macrophages and lymphocytes. Increased Th17 infiltration led to the further secretion of IL17 to upregulate the hepatic expression of macrophage and lymphocyte chemokine CXCL10 expression, thus forming a positive loop to enhance immune cell infiltration [71,72]. In a high-fat diet (HFD)-induced NAFLD mouse model, IL-17A and IL17-RA expression increased after HFD challenge; in comparison, the knockout of IL17-RA increased steatosis but decreased steatohepatitis, suggesting a central role of the IL-17 axis in the development and progression of NAFLD to steatohepatitis [73]. Hepatic stellate cells (HCSs) play a key role in the initiation of liver fibrosis. Thomas Fabre et al. showed in vitro that IL17A upregulates the HSCs surface expression of TGF-βRII and enhances their response to TGFβ, thus promoting steatohepatitis, indirectly emphasizing the importance of Th17 in NASH progression [74]. Notably, Th17 also secretes cytokines IL22 and IL23, which primarily play a protective role in NASH initiation and progression. As the primary origin of IL22 is Th22, the function of IL22 will be discussed in the Th22 section.

***Th22*:** Activation of the transcription factor aryl hydrocarbon receptor (AhR) can differentiate naïve CD4^+^T cells into Th22 cells and promote Th22 IL-22 secretion [75]. Interleukin-22 (IL-22) is the main cytokine secreted by Th22, and RORγt is a positive regulator of Th22 differentiation, whereas T-bet negatively regulates Th22 polarization [76]. IL-22 is associated with inflammatory histopathology, maintains intestinal homeostasis through multiple mechanisms, and plays a role in host defense against pathogens [77]. Xiaoting Wang et al. found that the induction of IL-22 from naïve CD4^+^T cells is impaired in obese mice. Exogenous IL-22 administration significantly reverses many of the metabolic symptoms, including hyperglycemia and insulin resistance, found in genetically obese mice and mice fed a high-fat diet [78]. In hepatitis, IL-22 counteracts the destructive immune response to limit liver tissue damage [79]. An in vitro model of hepatocyte lipotoxicity induced by palmitate treatment showed that IL-22 reduces palmitate-induced lipid toxicity through the PI3K-mediated inhibition of JNK in hepatocytes; however, in the presence of IL17, the protective role of IL22 in lipid toxicity was found to have disappeared [44]. Long-term treatment with IL-22 decreases the expression of enzymes involved in lipid synthesis and the elongation of fatty acids; it also lowers the levels of triglycerides and cholesterol in the liver [80]. In addition, IL-22 inhibits apoptosis and increases the expression of genes associated with anti-apoptosis [81].

In NASH livers, IL-22 induces metallothionein to block hepatic oxidative stress and inhibit the activation of oxidative stress-associated stress kinases. IL-22 treatment attenuates the inflammatory function of hepatocyte-derived mitochondrial DNA-enriched extracellular vesicles, thereby suppressing liver inflammation in NASH [82]. In HFD-induced hepatic steatosis, IL-22 also plays a protective role by regulating hepatic lipid metabolism [80]. In conclusion, IL-22 plays a protective role in liver fibrosis and inhibits the progression of NASH.

***Treg*:** Regulatory T cells (Tregs) make up a key subgroup of CD4^+^T cells playing an important regulatory role in maintaining immune tolerance and homeostasis via the secretion of immunosuppression cytokines, including IL10 and IL33 [83,84]. It has been reported that HFD-induced steatosis is associated with the depletion of hepatic Tregs, which may be the result of the lower expression of Bcl-2 by Tregs and increased susceptibility to oxidative stress-induced apoptosis. Treatment of mice with the antioxidant Mn (III) tetras (4-benzoic acid) porphyrin chloride was found to reduce Treg apoptosis and liver inflammation in HFD-induced steatosis, implying that Treg apoptosis facilitates the transformation of simple steatosis into steatohepatitis [85]. The preventative role of Tregs in NASH development was further confirmed by an MCD diet-induced NASH model in which Yoon Seok Roh et al. showed that Treg-depleted mice displayed aggravated steatohepatitis compared to wild-type mice. In contrast, in NASH-inflamed hepatic tissue, increased TNF-α and type 1 IFNs could induce hepatocyte death and inhibit the activity of Tregs, promoting Treg apoptosis to enhance NASH progression [86].

The antifibrotic effect of Tregs on NASH resolution is due to a certain extent to the immunosuppressive effect of the cytokines, such as IL-10, secreted by Tregs [87]. However, Tregs also secrete profibrotic cytokines, such as TGF-β, which is considered to be an important factor in the development of steatosis and fibrosis [88]. Therefore, Tregs may have a dual effect on NASH. Indeed, Treg-derived Areg has been reported to promote tissue repair, including in the heart [89] and skeletal muscle repair after damage [90]. Using a choline-deficient, L-amino acid-deficient high-fat diet (CDAA–HFD) model of NASH, Thomas M. Savage et al. showed that the number of Tregs was increased in CDAA–HFD-induced NASH livers. Importantly, by using human samples, they were also able to show that the proportion of Tregs in human NASH liver samples increased compared with normal hepatic tissue, which is consistent with the fact that hepatocyte injury contributes to the enrichment of Tregs within inflamed and damaged tissues, not solely in steatotic livers. The specific deletion of Areg in Tregs protected the mice from CCL4-induced fibrosis [91]. Mechanistically, Areg is secreted by NASH-infiltrating Treg-activated profibrotic transcriptional programs in hepatic stellate cells via epidermal growth factor receptor (EGFR) signaling [91]. Therefore, the function of Tregs in NASH depends on Treg cytokine secretion and hepatic tissue inflammation status. Future studies are needed to further uncover the effects of subpopulations of Tregs on NASH progression using new technologies, such as single-cell RNA sequencing.

## 3. Immunomodulatory Role of CD4^+^T Cells in NASH–HCC Transition and HCC Progression

***NASH–HCC transition*:** In an MYC-ON MCD NAFLD mouse model, Chi Ma et al. showed that the disruption of mitochondrial function by linoleic acid from NAFLD liver caused oxidative damage and mediated the selective loss of intrahepatic CD4^+^T lymphocytes but not of CD8^+^T lymphocytes. Loss of intrahepatic CD4^+^T lymphocytes accelerated tumor development, implying that NAFLD-induced CD4^+^T cell loss promotes NAFLD–HCC transition [92]. Furthermore, the same group demonstrated that NAFLD/NASH-associated steatohepatitis reduced the ability of immunotherapeutic agents to inhibit intrahepatic transplanted melanoma or colon cancer tumor growth by reducing the number of tumor-infiltrating CD4^+^T cells. The addition of N-acetylcysteine (NAC), meanwhile, prevented CD4^+^T cells loss and restored immunotherapy efficiency, indicating the necessity for CD4^+^T cells in HCC immunotherapy [93]. Hepatocyte apoptosis is a well-defined form of cell death in NASH and is considered the primary cause of liver inflammation and fibrosis. Zhigang Lei et al. demonstrated in human and animal models that hepatocyte CD1d expression in hepatocytes protected against hepatocyte apoptosis and alleviated hepatic inflammation and injuries in NASH [94]. Similarly, Ritesh Kbaboota et al. used liver biopsies to demonstrate that hepatic cell senescence is strongly related to NAFLD/NASH severity. Moreover, they found that BMP4 expression is antisenescent, while Gremlin 1 is prosenescent and antagonistic to BMP4 [95]. By using transgenic mouse models, Tae-Won Kang et al. showed that CD4^+^T cells participate in the immune-mediated clearance of premalignant senescent hepatocytes. Therefore, defects in senescence surveillance performed by CD4^+^T cells as well as by macrophages, could result in the development of murine hepatocellular carcinomas [96], which provides another mechanism governing how CD4^+^T cells affect NASH–HCC transition. Together, these results imply the protective role of CD4^+^T in NASH–HCC transition via immunosurveillance and the clearance of senescent hepatocytes. Contrary to the MYC-driven MCD diet NAFLD–HCC mouse model, Dominik Pfister et al. used a CD–HFD-induced NASH mouse model to show that, compared to prophylactic treatment involving anti-PD-1, which was able to increase CD–HFD-induced NASH–HCC transition with a greater number of tumor nodules and greater tumor size, a combination of anti-PD1 and anti-CD4 significantly decreased the number of HCC tumor nodules and the tumor size. These findings suggest that CD4^+^T cells promote NASH–HCC transition. Notably, in this mouse model, prophylactic treatment of anti-CD4 did not affect NASH–HCC transition [15]. The conflicting results underscore the NASH microenvironment in HCC transition, as well as the requirement for improved animal models of NASH–HCC with high fidelity to human pathogenesis [24,97].

Even though the number of hepatic CD4^+^T cells declines in NASH-associated HCC, the subpopulation of Th17 cells, which have been shown to support NASH progression, increases in number during NASH–HCC transition [98]. By using *IL-17RA*−/−mice, the authors of one study reported that IL-17RA signaling regulated liver injury in the progression of NAFLD; the neutralization of IL-17A, meanwhile, significantly reduced obesity-driven hepatocellular damage. The above findings position the IL-17 pathway as a possible therapeutic target in NAFLD for steatohepatitis progression [73]. Hepatic unconventional prefoldin RPB5 interactor (URI) expression induces DNA damage, which triggers an increase in IL-17A levels and upregulation of Th17 cell infiltration. Blocking IL-17A with antibodies or genetic ablation of the IL-17A receptor in myeloid cells prevents NASH and HCC [99]. In one study, fibroblast growth factor 21 (FGF21) attenuated TLR-4-triggered hepatocyte IL-17A expression, and anti-IL-17A treatment alleviated HCC nodule numbers in NASH mice. These findings provide further confirmation of the critical role of IL-17A in NASH progression and NASH–HCC transition, indirectly implying the oncogenic role of Th17 in NASH-associated HCC [100].

Tregs have been widely studied as immunosuppressive immune cells for use in cancer surveillance. In a study focusing on NASH, although the absolute number of CD4^+^T cells decreased compared to that in normal hepatic tissue, the number of Tregs was selectively increased by neutrophils via neutrophil extracellular trap (NET)-mediated metabolic reprogramming of naïve CD4^+^T cells. Since Tregs can suppress immunosurveillance in the premalignant stages of HCC, therapies targeting NETs and Tregs dramatically inhibit NASH–HCC transition [101].

***HCC progression*:** In a study involving 39 matched tissue samples from HCC, non-tumor, and leading-edge specimens of 13 HCC patients, using single-cell-scaled time-of-flight mass cytometry (CyTOF), it was shown that the number of CD4 effector memory T cells (Tems) and Tregs gradually increased from the non-tumor region to the HCC tumor region [102]. Nada Chaoul et al. used flow cytometry to analyze HCC tumors, peritumor tissue, and peripheral blood mononuclear cells from 14 patients and found that, compared to the peritumor region, the recruitment of cytotoxic cells including CD4^+^ and CD8^+^T cells in the tumor itself was impaired; in contrast, the effector memory CD4^+^T cells were more attracted to HCC tissue. In addition, CD4^+^T cells in HCC tumors showed a higher degree of activation than their circulating counterparts, but also showed a more exhausted phenotype [103]. Using transgenic mouse models of MYC-induced T cell acute lymphoblastic lymphoma and pro-B cell leukemia, Kavya Rakhra et al. described how CD4^+^T cells home to the tumor and are sufficient to induce sustained tumor regression upon MYC inactivation [104]. In a later study involving a MYC-driven HCC mouse model, they showed that depletion of MYC mRNA and protein expression via MYC antisense oligonucleotides led to decreased HCC cancer cell proliferation, increased cancer cell apoptosis and senescence, and remodeling of the tumor microenvironment via the recruitment of CD4^+^T cells, highlighting the antitumor effect of CD4^+^T cells in HCC tumor regression [105]. In another report, Chunxing Zheng et al. showed that tumor antigens drive the clonal expansion of choline acetyltransferase (ChAT)-expressing CD4^+^T cells to curtail the development of liver cancer by supporting antitumor immune responses. Mechanistically, the cholinergic activity intrinsic in T cells constrains Ca2+-NFAT signaling induced by T cell antigen receptor engagement. CD4^+^T cell-specific ablation of Chat caused an alteration in Tregs and induced PD-1 inhibitory activity, which led to compromised antitumor immunity [106]. In our own experiments, knockout of Bcl6 in a mouse liver cancer cell line promoted tumor immune rejection and anti-PD-1 immunotherapy efficiency. Interestingly, the depletion of CD4^+^T cells (but not CD8^+^T cells) eliminated the immune rejection toward Bcl6 knockout HCC. Taken together, this evidence confirms the role of CD4^+^T cells in HCC immunosurveillance.

Tregs, another subgroup of CD4^+^T cells, are widely studied as immunosuppressive cells in cancer immunology. Exosomal circGSE1, derived from HCC cells, could induce the expansion of Tregs by regulating the miR-324-5p/TGFBR1/Smad3 axis, and thus serves as a promising biomarker for HCC immunotherapy [107]. p38-MAX signaling in circulating tumor cells (CTCs) derived from HCC upregulates the expression of CCL5, while the expression of CCL5 recruits Tregs to facilitate CTC immune escape and metastatic seeding (Table 1) [108].

## 4. Implications of CD4^+^T Cells in the Treatment of NASH, NASH–HCC Transition, and HCC Immunotherapy

***NASH*:** At present, treatment options for NASH remain limited. In March 2024, the U.S. Food and Drug Administration approved Rezdiffra (resmetirom), the first drug used to treat adult patients with noncirrhotic nonalcoholic steatohepatitis (NASH) and moderate to advanced liver scarring (fibrosis). In a trial comparing bariatric metabolic surgery with lifestyle intervention plus optimal medical care for NASH patients, bariatric metabolic surgery was found to be more effective than lifestyle intervention and optimized medication in the treatment of NASH [110]. In the previous sections of this paper, we demonstrated that CD4^+^T cells play an essential role in NASH progression; however, the treatment of NASH via the targeting of CD4^+^T cells is still very limited in application. Using mouse models, Ravi P Rai et al. showed that blocking the integrin receptor αβ-mediated recruitment of CD4^+^T cells to the gut and liver not only reduces liver inflammation and fibrosis but also improves symptoms of the metabolic disorders associated with NASH, suggesting that integrin receptor αβ may be a potential treatment target for NASH [111]. As previously discussed, neutralization of IL-17A significantly reduced obesity-driven hepatocellular damage in a study cohort; therefore, targeting Th17 might also be a possible therapeutic strategy for NASH [73]. Conclusively, based on the important role of CD4^+^T cells in NASH initiation and NASH–HCC transition, more preclinical studies are required to explore the possibility of targeting CD4^+^T cells to inhibit NASH progression.

***NASH–HCC transition*:** At present, there are no therapeutic modalities that target the NASH–HCC transition process. However, in a pre-clinical study, Chongshu Jian et al. found that at an equivalent dose of less than one-tenth of the current clinical dose used for HCC treatment, sorafenib administration successfully prevented NASH–HCC transition in both mice and monkeys without any observed significant adverse events. In addition, it was found that the above low-dose sorafenib treatment also protects against diet-induced NASH in mice and improves the main features of NASH, including hepatic steatosis, inflammation, and fibrosis. Mechanistically, this benefit of sorafenib in NASH treatment involves the induction of mild mitochondrial uncoupling and the activation of AMP-activated protein kinase [112]. Using NASH-related HCC models, Kohe Yamada et al. showed that HIF-1α levels increased and that this increase promoted tumor aggressiveness and the inflammatory response. Tipifarnib, an arnesyltransferase inhibitor, strongly suppressed HIF-1α upregulation and inhibited cancer cell proliferation while concurrently inducing cancer cell apoptosis, possibly via upregulation of ROS production. Moreover, in the NASH-driven HCC mouse model induced by a diethylnitrosamine (DEN) + choline-deficient, L-amino acid-defined, high-fat diet (CDAHFD), treatment with tipifarnib significantly ameliorated tumor nodule formation in association with decreased serum interleukin-6 levels [113].

In addition to the available evidence demonstrating the role of CD4^+^T cells in NASH progression and NASH–HCC transition, as reviewed in the previous sections of this paper, it was reported that NAFLD induces carnitine palmitoyltransferase (CPT) gene expression in intrahepatic CD4^+^T cells. Upregulation of carnitine palmitoyltransferase (CPT) expression increases mitochondrial reactive oxygen species (ROS) production, which leads to CD4^+^T cell apoptosis and the promotion of NAFLD–HCC transition. Furthermore, treatment with the CPT inhibitor perhexiline reduced CD4^+^T apoptosis and slowed the development of NAFLD-associated HCC [114]. In a mouse model involving the administration of a high-fat diet (HFD), the green tea extract Theaphenon E (TE) significantly inhibited lipid accumulation, attenuated NAFLD symptoms, improved the survival of CD4^+^T cells, and significantly prevented the occurrence of NAFLD-related HCC [115].

***NASH–HCC immunotherapy*:** Since early-stage HCC is clinically asymptomatic, when a diagnosis of HCC is finally made, patients are always in the middle or advanced stages of the disease, making surgical resection and local ablative therapy impossible to perform. Receptor tyrosine kinase inhibitors, including sorafenib and levatinib, are first-line treatments for systemic HCC therapy. Of late, emerging T cell-based treatment modalities have shown great potential in HCC treatment. The combination of anti-PD-L1 (atezolizumab) and anti-VEGF mAb bevacizumab has proven to be superior to sorafenib in terms of overall survival and progression-free survival. The objective response rate is a landmark of immunotherapy for HCC, hence its inclusion as the first approved first-line treatment immune checkpoint blockade therapy for HCC [14]. A few years later, in November 2022, the FDA approved a new combination of anti-PDL1 mAb durvalumab and anti-CTLA4 mAb tremelimumab, which provided a 4-year OS rate of 25.2% for patients with advanced HCC, as another first-line immunotherapy targeting immune checkpoint [116]. In addition to immune checkpoint blockade, chimeric antigen receptor-T (CAR-T) cell therapy has become another appealing immunotherapy modality, even though its use for disease treatment is only in the preclinical stage of testing. Glypican-3 (GPC3) is a well-characterized HCC-associated antigen that has been shown to be a promising target for HCC CAR-T therapy [117,118]. In preclinical studies, cancer vaccines based on tumor-associated antigens, such as alpha-fetoprotein (AFP), Glypican-3, multidrug resistance-associated protein 3 (MRP3), etc., have also shown some effects for use in HCC treatment [119].

Notwithstanding the clinical benefit of immune checkpoint blockade therapy in HCC, the response rate of atezolizumab in combination with bevacizumab is only around 30%. Employing a meta-analysis, Dominik Pfister et al. showed that in three large, randomized phase III trials (CheckMate-459, IMbrave150, and KEYNOTE-240) involving patients with advanced HCC, an immune checkpoint blockade improved the survival of viral-induced HCC patients but not non-viral HCC patients, thus highlighting the importance of HCC patient stratification according to HCC etiology. Furthermore, the researchers used clinical trials to show that the treatment of NAFLD-associated HCC patients treated with anti-PD(L)1 was associated with shortened median overall survival compared to patients with other etiologies. Moreover, using preclinical models of NASH-induced HCC, they showed that prophylactic treatment of anti-PD1 led to an increase in the incidence of NASH–HCC and in the number and size of tumor nodules. However, the increase in HCC triggered by anti-PD1 treatment was prevented by depletion of CD8^+^T cells or TNF neutralization, suggesting that CD8^+^T cells help induce NASH–HCC rather than invigorating or executing immune surveillance. The above results indicate that NASH-associated HCC patients did not benefit from checkpoint inhibition therapy, probably owing to NASH-related aberrant T cell activation causing tissue damage, leading to impaired immune surveillance [15].

Immune checkpoint inhibitors, such as anti-PD(L)1, restore T cell function and infiltration in the tumor TME [120]. However, patients suffering from NASH-associated HCC were found to be unable to benefit from this treatment modality. In studies on NASH-associated HCC, CD4^+^T cells were found to play different roles. First, CD4^+^T cells promote NASH progression by inducing hepatocyte apoptosis via the secretion of IFN-γ [46,47]; conversely, CD4^+^T cells also play an immunosurveillance role in HCC initiation and progression [106]. Thus, in addition to CD8^+^T cells, which are widely regarded as cytotoxic T lymphocytes, the function and mechanism governing how CD4^+^T cells modulate NASH-associated HCC immunotherapy efficiency warrant further attention.

## 5. Conclusions

**Figure 2:**
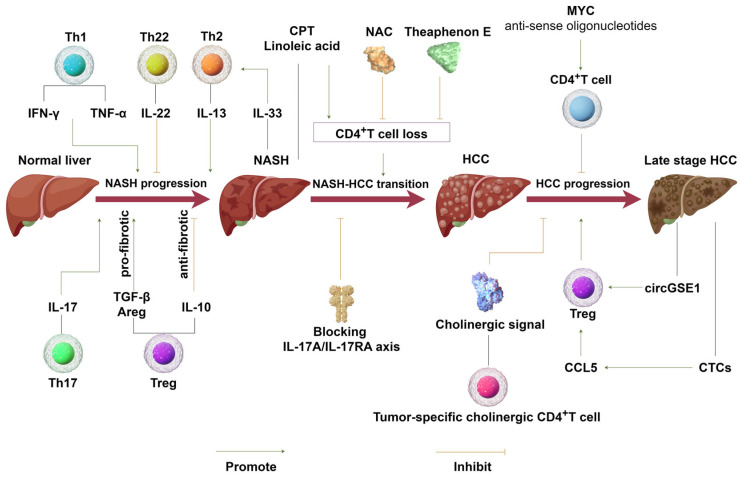
The role of CD4^+^T cells in NASH and hepatocellular carcinoma. During NASH progression, the function of CD4^+^T cells depends on the subtype, as well as on the secreted cytokines. NASH increases CD4^+^T cell apoptosis via the upregulation of CPT expression, or linoleic acid-induced oxidative damage promotes NASH–HCC transition. Treatment with NAC and Theaphenon E was found to inhibit CD4^+^T cell apoptosis, thus inhibiting NASH–HCC transition. CD4 effector T cells suppress HCC progression through cholinergic activity and immune suppressive mechanisms; in contrast, CTCs and circGSE1 derived from HCC upregulate Treg infiltration to promote HCC progression.

Due to the increasing worldwide epidemic of obesity, NASH has become one of the leading causes of hepatocellular carcinoma. In NASH development, CD4^+^T cells function differently depending on their subpopulation differentiation. Th1, Th2, and Th17 promote NASH progression, while Th22 ameliorates NASH damage. Tregs could prevent NASH via their immunosuppression mechanism; moreover, they also play a profibrotic role by secreting factors such as amphiregulin (Figure 2). The conflicting role of CD4^+^T cells has also been reported in NASH–HCC transition, possibly because of the different mouse models used, highlighting the importance of improved animal models of NASH-induced HCC with high fidelity to human pathogenesis.

HCC immunotherapy, particularly immune checkpoint blockade therapy, has been approved as the first-line treatment for HCC; however, NASH-associated HCC patients respond much less efficiently than viral-induced HCC patients and are considered a predictor of unfavorable outcomes. Thus, further studies are required to examine the underlying mechanism governing how NAFLD or NASH-associated HCC resists an immune checkpoint blockade-induced pro-inflammatory response. Based on the important role of CD4^+^T cells in NASH initiation, progression, and NASH–HCC transition, the role of CD4^+^T cells in HCC immune checkpoint blockade efficiency warrants the acquisition of further evidence.

## Figures and Tables

**Table 1:** Function of CD4^+^T cells in NASH and HCC progression.

Process	Subset	Effect	Mechanism	Model	References
NASH progression	Th1	Promote	IFN-γ and TNF-α	NASH and NAFLD patient samples, MCD, MCD-HF	[36,44,50]
	Th2	Promote	IL-33	NASH and NAFLD patient samples	[36,62]
	Th17	Promote	IL-17	MCDD, HFD	[71,72,73]
	Th22	Inhibit	IL-22	HFD	[80,82,109]
	Treg	Bidirectional	TGF-β and Areg (Promote) IL-22 (Inhibit)	MCD, CDAA-HFD	[88,90,91]
NASH–HCC transition	effector CD4^+^T	Inhibit	Immunological surveillance	MYC-ON MCD	[15,92]
	Th17	Promote	IL-17A/IL-17RA axis	---	[99,100]
	Treg	Promote	Immunosuppressive	---	[101]
HCC progression	ChAT-CD4^+^T	Inhibit	Cholinergic signal	---	[105]
	Treg	Promote	miR-324-5p/TGFBR1/Smad3 axis	---	[106,107]
